# Higher plasma levels of complement C3a, C4a and C5a increase the risk of subretinal fibrosis in neovascular age-related macular degeneration: Complement activation in AMD

**DOI:** 10.1186/s12979-016-0060-5

**Published:** 2016-02-16

**Authors:** Judith Lechner, Mei Chen, Ruth E. Hogg, Levente Toth, Giuliana Silvestri, Usha Chakravarthy, Heping Xu

**Affiliations:** The Wellcome-Wolfson Institute of Experimental Medicine, Queen’s University Belfast, 97 Lisburn Road, Belfast, BT9 7BL UK

**Keywords:** Age-related macular degeneration, Choroidal neovascularisation, Complement, Subretinal fibrosis

## Abstract

**Background:**

The aim of this study was to investigate the plasma levels of complement C3a, C4a, and C5a in different types of neovascular age-related macular degeneration (nAMD) and whether the levels were related to patients’ responsiveness to anti-VEGF therapy.

**Results:**

Ninety-six nAMD patients (including 61 with choroidal neovascularisation (CNV), 17 with retinal angiomatous proliferation (RAP), 14 with polypoidal choroidal vasculopathy (PCV) and 4 unclassified patients) and 43 controls were recruited to this case–control study. Subretinal fibrosis was observed in 45 nAMD patients and was absent in 51 nAMD patients. In addition, the responsiveness to anti-VEGF (Lucentis) therapy was also evaluated in nAMD patients. Forty-four patients were complete responders, 48 were partially responders, and only 4 patients did not respond to the therapy. The plasma levels of C3a, C4a and C5a were significantly higher in nAMD patients compared to controls. Further analysis of nAMD subgroups showed that the levels of C3a, C4a and C5a were significantly increased in patients with CNV but not RAP and PCV. Significantly increased levels of C3a, C4a and C5a were also observed in nAMD patients with subretinal fibrosis but not in those without subretinal fibrosis. Higher levels of C3a were observed in nAMD patients who responded partially to anti-VEGF therapy.

**Conclusions:**

Our results suggest increased systemic complement activation in nAMD patients with CNV but not RAP and PCV. Our results also suggest that higher levels of systemic complement activation may increase the risk of subretinal fibrosis in nAMD patients.

**Electronic supplementary material:**

The online version of this article (doi:10.1186/s12979-016-0060-5) contains supplementary material, which is available to authorized users.

## Background

Neovascular age-related macular degeneration (nAMD), or wet AMD, is the leading cause of blindness in the elderly population and is characterised by the growth of abnormal blood vessels in the macular region of the retina. There are different subtypes of nAMD and the most commonly encountered is choroidal neovascularisation (CNV) which is characterised by the infiltration of abnormal neovascular complexes into the space between the retinal pigment epithelium and Bruch’s membrane or the subretinal space [1]. Neovascular complexes may also arise de novo from retinal vasculature known as retinal angiomatous proliferation (RAP) and these are known to fuse with CNV [2]. Another subtype of neovascularisation, polypoidal choroidal vasculopathy (PCV), is characterised by a branching vascular network arising from the choroid with polypoidal lesions underneath the RPE [3]. VEGF is elevated in eyes of nAMD patients and plays an important role in the neovascularisation process and vascular permeability in nAMD and intravitreal injection of anti-VEGF antibody is the standard care for nAMD [1].

The pathogenesis of AMD is complex and incompletely understood with genetic as well as clinical and environmental factors (such as age, family history of AMD, cardiovascular disease, body mass index and cigarette smoking) known to influence the risk of developing this disease, however, the underlying mechanisms remain elusive [4–6]. Compelling evidence suggests that inflammation plays a critical role in the aetiology of AMD [7, 8] and a number of studies have specifically highlighted the role of the complement system in AMD. Many of the genetic variants that have been associated with AMD lie in genes involved in the complement cascade, including complement factor H (CFH) [9], complement component 3 (C3) [10], complement component 2 (C2) and complement factor B (CFB) [11]. A number of studies have shown increased levels of complement expression in the maculae of AMD patients [12, 13]. Complement fragments, including C3a and C5a [14], and the membrane attach complex (MAC or C5b-9) [15] were found in drusen of patients with AMD as well as complement activating proteins such as amyloid beta [16] and lipofuscin [17]. Besides the local complement activation in AMD, systemic complement activation has also been detected in patients with AMD. Increased serum levels of complement fragments (e.g. Ba, C3d) [18–20] and changes in the expression of complement regulatory proteins (e.g. CD46, CD59) [21] have been reported in AMD. Complement activation and accumulation of MAC has been found in choriocapillaris, which are part of the systemic circulation, during normal aging and especially in patients with AMD [22]. It is clear that uncontrolled or dysregulated complement activation may contribute to macular lesion development in AMD, which offers the opportunities for complement-targeted immune therapy. Indeed a number of complement inhibitors are in phase I, II and III clinical trials for AMD [23, 24]. In view of the diversity of AMD phenotype, it is likely that different immune mechanisms may be involved in different types of AMD, and this is exemplified by the diverse response to anti-VEGF therapy observed in various clinical studies [25]. Therefore, it is important to understand which type(s) of AMD is associated with uncontrolled complement activation.

The complement system can be activated through the classical pathway (CP), the mannose-binding lectin (MBL) pathway and the alternative pathway (AP) [26]. Complement fragments C3a and C5a can be generated by any of these activation pathways, whereas C4a is generated when the CP or MBL pathway is activated. Elevated levels of these complement fragments are indicatives of increased complement activation. In this study, we measured the plasma levels of C3a, C4a and C5a in nAMD patients and correlated the expression levels with clinical presentations as well as the responsiveness to anti-VEGF therapy. Ninety-six nAMD patients (including 61 CNV, 17 RAP, 14 PCV and 4 unclassified patients) and 43 controls were recruited to this case–control study.

## Results

### Clinical evaluation

Of the 139 study participants, 43 were controls and 96 had diagnosed nAMD. Despite our efforts to recruit age matched controls, there was a significant difference in age between controls and nAMD patients (*P* = 0.002) as shown in Table 1. There were no significant differences regarding gender distribution, family history of AMD, history of cardiovascular disease, history of hypertension, history of diabetes, BMI and smoking habits between controls and nAMD patients. There were more patients taking vitamins and low-dose aspirin compared to controls (*P* = 0.011 and 0.001 respectively) (Table 1).

**Table 1:** Demographic and clinical characteristics of nAMD patients and controls

	All	Controls	nAMD	*P* value
	(*n* = 139)	(*n* = 43)	(*n* = 96)	nAMD vs Control
Age (median (range)), years	78.6 (53–93)	74.0 (58–92)	80.1 (53–93)	**0.002** ^a^
Female (number (%))	63 (45)	19 (44)	44 (46)	1.000^b^
Family history of AMD (number (%))	32 (23)	6 (14)	26 (27)	0.127^b^
History of cardiovascular disease (number (%))	36 (26)	9 (21)	27 (28)	0.528^b^
History of hypertension (number (%))	81 (58)	23 (53)	58 (60)	0.576^b^
History of diabetes (number (%))	15 (11)	2 (5)	13 (14)	0.150^b^
Body Mass Index (mean ± SD)	26.1 ± 4.4	26.1 ± 5.1	26.0 ± 4.0	0.980^c^
Smoking status				0.381^b^
Non-smoker (number (%))	56 (40)	20 (47)	36 (38)	
Former smoker (number (%))	70 (50)	20 (47)	50 (52)	
Current smoker (number (%))	12 (9)	2 (5)	10 (10)	
Taking cardiovascular medication (number (%))	102 (73)	28 (65)	74 (77)	0.212^b^
Taking vitamins (number (%))	28 (20)	3 (7)	25 (26)	**0.011** ^b^
Taking low-dose aspirin (number (%))	43 (31)	5 (12)	38 (40)	**0.001** ^b^

^a^Mann Whitney *U* test

^b^Pearson’s chi-square test

^c^Independent samples *t*-test

*SD* standard deviation

Bold *P* < 0.05

The average duration between the last anti-VEGF treatment and the day of blood collection was 140.1 ± 223.8 days (interquartile range: 42.0–127.0 days). No participant had received anti-VEGF treatment within 4 weeks prior to blood collection. The average number of anti-VEGF injections received per nAMD patient prior to blood collection was 16.1 ± 10.7 (interquartile range 8.3–21.8). There was no correlation between the number of anti-VEGF injections received and the concentration of C3a (Pearson's correlation coefficient (r) = 0.08; *P* = 0.448), C4a (r = −0.03; *P* = 0.771) and C5a (r = −0.16; *P* =0.131) (see Additional file 1).

### Complement fragments in nAMD patients and controls

All three complement fragments, C3a, C4a and C5a were significantly increased in nAMD patients compared to controls in the univariate analysis (*P* < 0.001, *P* = 0.005 and 0.049 respectively) and these associations remained significant in the multivariate analysis after correction for age and gender (*P* = 0.001, 0.012 and 0.045 respectively) (Table 2). In the nAMD group, C3a and C4a were significantly increased in those with a family history of AMD (*P* = 0.058 and 0.011 respectively) compared to those without a family history of AMD. The plasma level of C5a was not associated with a family history of AMD. Due to this association, we included the confounder “family history of AMD” in the multivariate analysis.

**Table 2:** C3a, C4a and C5a plasma levels in nAMD patients and controls

			Univariate analysis	Multivariate analysis (age and gender)			Multivariate analysis (age, gender and family history of AMD)		
Variables (ng/ml)	Controls (mean ± SD) *n* = 43	nAMD (mean ± SD) *n* = 96	*P* value nAMD vs Controls^a^	*P* value nAMD vs Controls^b^	Odds ratio	95 % confidence interval for odds ratio	*P* value nAMD vs Controls^c^	Odds ratio	95 % confidence interval for odds ratio
C3a	11.95 ± 3.30	14.65 ± 4.48	**<0.001**	**0.001**	241.44	8.44–6910.14	**0.005**	140.67	4.53–4372.57
C4a	68.91 ± 33.23	108.48 ± 83.83	**0.005**	**0.012**	5.81	1.46–23.05	**0.031**	4.79	1.15–19.88
C5a	8.34 ± 2.05	9.43 ± 2.73	**0.049**	**0.045**	20.55	1.07–396.06			

^a^Independent samples *t*-test

^b^Multinomial logistic regression corrected for age and gender

^c^Multinomial logistic regression corrected for age, gender and family history of AMD

Bold *P* < 0.05

*SD* standard deviation

When comparing nAMD patients and controls, after including the confounder “family history of AMD” in the multivariate analysis for C3a and C4a, the increase in C3a and C4a in the nAMD group remained significant (*P* = 0.005 and 0.031 respectively) as shown in Table 2. The concentrations of complement components were not associated with vitamin or low dose aspirin intake or any other confounders.

### Complement fragments in patients with CNV, RAP and PCV

Out of the 96 nAMD participants, 61 had CNV, 17 with RAP, 14 with PCV and 4 were unknown. There were no significant differences in complement components when comparing between neovascular AMD subtypes, although there was a trend for higher C3a, C4a and C5a concentrations in those classified as CNV. On comparing subgroups of nAMD participants with controls, there was a significant increase in C3a, C4a and C5a in those with CNV when compared to controls in the univariate analysis (*P* < 0.001, *P* = 0.003 and 0.044 respectively; Table 3) and these associations remained significant in the multivariate analysis after correction for age and gender as well as family history of AMD for C3a and C4a (*P* = 0.001, 0.009 and 0.008 respectively). There were no significant differences in the plasma levels of C3a, C4a, and C5a between RAP or PCV versus controls (Table 3).

**Table 3:** Univariate analysis of C3a, C4a and C5a plasma levels in nAMD patients with CNV, RAP and PCV

Variables (ng/ml)	Controls (mean ± SD)	CNV (mean ± SD)	RAP (mean ± SD)	PCV (mean ± SD)	*P* value controls vs CNV vs RAP vs PCV^a^	*P* value
						Bonferroni *post hoc* test
	*n* = 43	*n* = 61	*n* = 17	*n* = 14		
C3a	11.95 ± 3.30	15.29 ± 4.29	14.15 ± 5.93	12.55 ± 2.86	**<0.001**	**<0.001** ^b^
						0.609^c^
						1.000^d^
C4a	68.91 ± 33.23	118.16 ± 93.41	84.73 ± 55.07	96.51 ± 63.71	**0.004**	**0.003** ^b^
						1.000^c^
						1.000^d^
C5a	8.34 ± 2.05	9.92 ± 2.81	8.10 ± 2.53	8.68 ± 1.95	**0.016**	**0.044** ^b^
						1.000^c^
						1.000^d^

^a^One-way ANOVA

^b^Controls vs CNV

^c^Controls vs RAP

^d^Controls vs PCV

Bold *P* < 0.05

*SD* standard deviation

### Complement fragments and subretinal fibrosis

Subretinal fibrosis was present in 45 (47 %) of nAMD patients (Table 4). When comparing complement fragments in patients with and without fibrosis to controls, C3a was significantly increased in both groups compared to the controls in the univariate analysis (*P* = 0.046 and <0.001 respectively; Table 4). After adjustment for age, gender and family history of AMD the difference in C3a between controls and patients with fibrosis remained highly significant (*P* = 0.001), whereas the difference between controls and patients without fibrosis was insignificant (*P* = 0.055). C4a was significantly increased in participants with fibrosis when compared to controls in the univariate (*P* = 0.003; Table 4) and multivariate analysis (*P* = 0.010). For C5a, the univariate analysis did not detect any significant differences between participants with or without fibrosis when compared to controls, however in the multivariate analysis there was a significant difference in C5a when comparing participants with fibrosis to controls (*P* = 0.018).

**Table 4:** Univariate analysis of C3a, C4a and C5a plasma levels in participants with nAMD with and without subretinal fibrosis

Variables (ng/ml)	Controls (mean ± SD)	Fibrosis absent (mean ± SD)	Fibrosis present (mean ± SD)	*P* value	*P* value	*P* value
	*n* = 43	*n* = 51	*n* = 45	Fibrosis absent vs present^a^	Fibrosis absent vs present vs controls^b^	Bonferroni *post hoc* test
C3a	11.95 ± 3.30	13.68 ± 3.05	15.75 ± 5.51	0.051	**<0.001**	**0.046** ^c^
						**<0.001** ^d^
C4a	68.91 ± 33.23	93.95 ± 59.44	124.94 ± 103.14	0.102	**0.004**	0.258^c^
						**0.003** ^d^
C5a	8.34 ± 2.05	9.14 ± 2.66	9.75 ± 2.80	0.343	0.088	

^a^Independent samples *t*-test

^b^One-way ANOVA

^c^Controls vs Fibrosis absent

^d^Controls vs fibrosis present

Bold *P* < 0.05

*SD* standard deviation

### Complement fragments and responsiveness to anti-VEGF therapy

Of the 96 nAMD patients, 44 (46 %) responded completely to the anti-VEGF therapy, 48 (50 %) partially responded to the therapy and 4 patients (4 %) did not respond to the therapy. Due to the limited number of non-responders in this study, this group was not included in the statistical analysis. When comparing the concentration of plasma complement fragments between partial and complete responders, we found a significant increase in C3a in partial responders in the univariate analysis (*P* = 0.041; Table 5) and the difference remained significant in the multivariate analysis after correcting for age, gender and family history of AMD (*P* = 0.033). No significant differences in plasma levels of C4a and C5a were identified when comparing complete responders with partial responders as shown in Table 5. The response to anti-VEGF therapy was not associated with the presence of subretinal fibrosis (*P* = 0.682; Pearson’s chi-square test).

**Table 5:** Univariate analysis of C3a, C4a and C5a plasma levels in nAMD patients not responding, partially responding and completely responding to anti-VEGF Ab therapy

Variables (ng/ml)	Controls (mean ± SD)	Non responders (mean ± SD)	Partial responders (mean ± SD)	Complete responders (mean ± SD)	*P* value
	*n* = 43	*n* = 4	*n* = 48	*n* = 44	Partial vs complete responders^a^
C3a	11.95 ± 3.30	13.97 ± 3.94	15.61 ± 5.18	13.67 ± 3.45	**0.041**
C4a	68.91 ± 33.23	79.97 ± 75.52	120.85 ± 98.30	97.57 ± 64.80	0.147
C5a	8.34 ± 2.05	8.62 ± 2.47	9.31 ± 2.72	9.63 ± 2.80	0.654

^a^Independent samples *t*-test

Bold *P* < 0.05

*SD* standard deviation

## Discussion

In the present study we report that complement fragments C3a, C4a and C5a are significantly elevated in the plasma of nAMD patients when compared to controls. Hence our results confirm previous findings of increased systemic complement activation in nAMD [18, 19]. The systemic levels of C3a, C4a and C5a in different types of nAMD (e.g. CNV, RAP, PCV and fibrosis) or in different anti-VEGF therapy responder groups have not been investigated before, and such studies are important as different immunomechanisms may be involved in different types of nAMD. In this study we found that higher plasma levels of C3a, C4a and C5a are associated with subretinal fibrosis and with CNV rather than RAP and PCV and that C3a was significantly increased in patients partially responding to anti-VEGF therapy.

How the complement system is activated in nAMD patients and how this may contribute to the development of nAMD is currently not well understood. Circulating complement fragments such as C3a and C5a may be recruited to the macula in AMD. Complement deposition (e.g., C3a, C5a, C5b-9) has been detected in RPE, Bruch’s membrane and choroid of AMD eyes [27]. Studies conducted in patient samples as well as in animal models of laser-induced CNV have shown that complement activation may contribute to CNV development at multiple levels. The membrane attack complex (MAC), the final product of complement activation, may directly induce CNV. C3-deficient mice are unable to form MAC and do not develop CNV after laser photocoagulation. In the same study, CNV was suppressed by inhibition of MAC formation through blockage of C3 or C6 [28]. In addition, MAC can upregulate pro-angiogenic factors such as VEGF, TGF-β2, and β-FGF in retinal cells [28, 29]. Furthermore, the expression of MAC is increased in choriocapillaris of the aging macula and the expression is further enhanced in patients with AMD [30].

Anaphylatoxins were also shown to be involved in experimental CNV. C3a and C5a were increased in the RPE/choroid after laser injury and blockage of C3a or C5a resulted in reduced CNV formation. CNV was reduced in mice deficient in C3a or C5a receptors or in mice treated with C3a or C5a receptor antagonist [14]. C3a and C5a were among the complement fragments detected in the RPE of nAMD patients and both can induce the expression of VEGF [14].

Systemic complement activation may also be involved in the activation of choroidal endothelial cells. C5a receptor was shown to be expressed in the endothelium of human choriocapillaris and treatment of human choroidal endothelial cells with C5a resulted in increased expression of ICAM-1, an intercellular adhesion molecule involved in leukocyte trafficking [31]. Furthermore, anaphylatoxins have been shown to be potent inflammatory mediators. Stimulation of human umbilical vein endothelial cells with C3a or C5a resulted in upregulation of pro-inflammatory cytokine production (e.g. IL-8 and IL-1β) [32]. C5a has been shown to promote IL-22 and IL-17 expression from human T-cells and both cytokines were shown to be increased in the serum of AMD patients [33]. In monocytes, C5a stimulation was shown to enhance LPS-induced production of IL-6 and TNFα [34]. Systemic complement activation may pre-condition leukocytes to a pro-inflammatory phenotype and may contribute to the local inflammatory responses in nAMD when recruited to the aging macula.

The role of C4a in nAMD development remains poorly elucidated [35] and deficiency of C4 did not affect CNV formation following laser photocoagulation in mice [36]. Nevertheless, in the present study we detected elevated plasma levels of C4a in nAMD patients, particularly in patients with CNV and in patients with subretinal fibrosis. Further studies are necessary to investigate the role of C4a in the pathogenesis of nAMD.

Subretinal fibrosis is a common finding in patients with late stage nAMD leading to considerable impairment of visual function although the underlying mechanisms remain poorly defined [1]. In this study we report significantly elevated plasma levels of C3a, C4a and C5a in patients with subretinal fibrosis. Systemic alterations in the complement system have previously been linked to subretinal fibrosis in nAMD. Singh et al. found reduced expression of complement regulatory protein CD46, which inhibits the production of C5a, on peripheral lymphocytes in nAMD patients with subretinal fibrosis [21]. C5a has been implicated in the development of fibrosis in a mouse model of chronic pancreatitis, where loss of C5 or injection of a C5a-receptor antagonist significantly reduced the level of pancreatic fibrosis [37]. Our results suggest uncontrolled complement activation and the formation of anaphylatoxins such as C3a, C4a and C5a may contribute to the development of subretinal fibrosis in nAMD and further studies are necessary to understand the detailed mechanisms.

We observed increased plasma C3a levels in patients partially responding to the anti-VEGF therapy when compared to patients completely responding to the therapy, suggesting C3a plasma level may be a biomarker in predicting outcomes to treatment. Biological plausibility for such an association exists in that C3a can induce the expression of other pro-angiogenic factors including IL-8 [38] which may explain why patients with increased levels of C3a respond less well to the anti-VEGF treatment. Furthermore, a SNP in the C3 gene has previously been linked to reduced central retinal thickness following anti-VEGF treatment [39], although subsequent studies have failed to replicate these findings [40]. Polymorphisms of complement factor H, C2 as well as interleukins IL-6 and IL-10 or VEGF genes are known to be linked to nAMD risk and may affect the pathogenesis of the disease [41]. These additional factors might also contribute to the responsiveness to anti-VEGF therapy [41].

Why C3a but not C4a and C5a is associated with responsiveness to anti-VEGF therapy is not known. The complement system can be activated through at least three pathways i.e., the CP, AP, and MBL pathway. C3a is generated when any of the pathways is activated, whereas the generation of C4a is restricted to the CP or MBL pathway and C5a to the terminal pathway. In addition, a recent study has shown that C3 can be cleaved into C3a and C3b intracellularly by cathepsin L (i.e., independently of C3 convertase) [42]. Therefore, changes in plasma levels of C3a, C4a and C5a may not follow the same pattern. The serum levels of C3a were similar in complete and non-responders, although only 4 patients were recruited to the non-responder group. Nevertheless we contend that confirming our results in a larger patient cohort and exploring the temporal associations between plasma levels of C3a and morphological changes after anti-VEGF therapy are likely to help elucidate the mechanisms of treatment responsiveness to these agents.

In this study, we also found significantly increased plasma levels of C3a, C4a and C5a in patients with CNV, but not RAP or PCV when compared to controls, suggesting that systemic complement activation may play a bigger role in CNV than in RAP or PCV. However, the results must be interpreted with caution as there were small numbers of participants in the RAP and PCV group (*n* = 17 and 14 respectively). Further studies using larger patient samples are necessary to confirm the results.

The strengths of the present study include independent grading of CNV type, fibrosis and anti-VEGF responsiveness, and systematic and extensive exploration of complement changes in different types of nAMD as well as in patients partially or completely responding to anti-VEGF treatment.

There are a number of limitations in the current study. Firstly, the number of controls enrolled in this study is relatively small and some of the analyses involved small groups of patients (i.e. patients with RAP *n* = 17, patients with PCV *n* = 14) with the attendant consequences of drawing conclusions based on small samples. Secondly, all participates were recruited from one location and the data only represent results from Northern Ireland population. Replication of the study findings in other locations and with much improved bigger numbers is necessary to confirm our results. Thirdly, there was a significant difference in age between nAMD patients and controls. Furthermore, patients were recruited at different times following diagnosis of nAMD. Consequently, some patients enrolled at an early stage of nAMD and classified as having no fibrosis, might still develop fibrosis during the course of the disease. Patients enrolled in this study were receiving anti-VEGF treatment prior to enrolment which may have altered systemic complement levels although there was no correlation with the number of anti-VEGF injections received and the levels of C3a, C4a or C5a. Furthermore the drug in use in our study site was ranibizumab which is cleared more rapidly from the systemic circulation and has the least effect on serum VEGF levels when compared to other anti VEGF agents [43]. Finally, the change of C3a and C5a in the whole study, while statistically significant, was small and the clinical and biological relevance of such a small difference warrants further investigations.

## Conclusions

In this study, we have demonstrated systemic complement activation in nAMD, particularly in patients with CNV or with subretinal fibrosis, and we have shown that higher plasma C3a levels are related to reduced responsiveness to anti-VEGF treatment. Our observations may have important implications in future management of nAMD. Complement inhibitors are currently in clinical trials for AMD [23, 24], and the identification of suitable patients is important for the success of this type of immune therapy. Our results provide evidence that nAMD patients with CNV and with subretinal fibrosis may benefit more from the complement inhibitor therapy compared to other subgroups of nAMD patients. Complement inhibitors may also be a supplement therapy for patients who partially respond to VEGF inhibitors.

## Methods

### Study participants

The study protocol was approved by the Research Ethics Committee of Queen’s University Belfast and procedures were performed in accordance with the tenets of the Declaration of Helsinki on research into human volunteers. Participants were recruited from the macular disease clinics in Belfast (Belfast Health and Social Care Trust, UK) with written informed consent obtained from every participant. Spouses, relatives or friends who accompanied patients and who were confirmed to be without retinal disease (fundus photography and optical coherence tomography (OCT)) were recruited as controls. All participants were older than 50 years of age and structured questionnaires were used to ascertain a history of medical conditions, current medication, family history of AMD, smoking habits (current, ex-smoker, never smoker) and body mass index (BMI). Participants with systemic inflammatory or autoimmune disorders (e.g. patients with active rheumatoid arthritis or active chronic bronchitis), participants undergoing steroid therapy or chemotherapy were excluded from the study.

The diagnosis of nAMD was by clinical examination and confirmed by multimodal imaging consisting of fundus photography, autofluorescence, optical coherence tomography, fluorescein angiography and Indocyanine green angiography. Participants were further subcategorised into CNV, RAP and PCV. Responsiveness to treatment was defined based on the participant achieving a fluid free macula at any stage during follow up. Participants were classified into the following 3 categories: Complete responder: Resolution of leakage at any point in time during follow up; Partial responder: Exhibiting dependence on VEGF inhibitors but a fluid free macula never achieved; Non responder: No morphological improvement or worsening.

### Sample collection

In this study most of the participants were receiving anti-VEGF therapy prior to enrolment. The number of anti-VEGF (ranibizumab, trade name Lucentis, Genentech, San Francisco, CA) injections received by each patient prior to blood collection was ascertained from the medical records. Peripheral blood samples were drawn in tubes containing ethylenediaminetetraacetic acid (EDTA) as an anticoagulant between 9:00 and 12:00 am and processed within three hours. The plasma was separated from the whole blood by centrifugation for 10 min at 300 *g*. The plasma fraction was collected and centrifuged again for 10 min at 2000 *g* to remove any residual cells and platelets before it was aliquoted and stored at −80 °C until analysis.

### Cytometric bead array

Complement fragments C3a, C4a and C5a were measured in the plasma by Cytometric Bead Array using a Human Anaphylatoxin Kit (BD Biosciences, Oxford, UK) according to the manufacturer’s instructions. In order to avoid spontaneous complement activation, plasma samples were thawed rapidly at 37 °C until just thawed and immediately transferred to ice. Plasma samples were diluted 1:20 with assay diluent prior to analysis.

Briefly, capture beads were mixed and incubated with diluted plasma samples and standards for 2 h at room temperature. After the incubation, samples were washed with wash buffer and incubated with anaphylatoxin PE detection reagent for 1 h at room temperature protected from light. After another washing step, samples were resuspended in wash buffer and fluorescence intensities were measured by flow cytometry (FACS CANTO II; BD Biosciences). Concentrations were calculated using the FCAP Array software version 3.0 (BD Biosciences, Oxford, UK).

### Statistical analysis

Statistical analysis was performed using the Statistical Package for the Social Sciences, Windows version 21 (SPSS Inc, Armonk, NY). Categorical demographic and clinical data were compared using Pearson’s chi-square test. The distribution of continuous variables was assessed for normality using the Kolmogorov-Smirnov test and logarithmic transformation was performed if necessary to achieve normal distribution. Normally distributed continuous samples were then compared using the Independent samples *t*-test or one-way ANOVA. Age was not normally distributed and the difference between controls and nAMD patients was analysed using the Mann–Whitney *U* test.

For the associations that were significant in the univariate analysis, multinomial logistic regression was performed to adjust for age and gender. All variables were also tested for association with family history of AMD, history of cardiovascular disease, history of hypertension, history of diabetes, smoking habits, BMI, taking of cardiovascular medication, vitamins and low-dose aspirin using the Independent samples *t*-test, one-way ANOVA or Pearson’s correlation. If significant associations were identified, adjustments were made in the multinomial logistic regression analysis. Pearson’s correlation was used to assess the correlation between the number of anti-VEGF injections a patient had received prior to blood collection and the concentration of complement components. Data were presented as mean ± standard deviation (SD) calculated from untransformed variables even if the statistical analysis was performed on transformed variables. *P* values <0.05 were considered statistically significant.

## Additional file

Additional file 1: Figure S1. The relationship between the number of anti-VEGF injections and the concentration of complement fragments in the plasma. No correlation was observed between the number of intravitreal anti-VEGF injections and the plasma levels of C3a, C4a and C5a. (PDF 118 kb)

## Abbreviations

AMD: Age-related macular degeneration
CNV: Choroidal neovascularisation
MAC: Membrane attack complex
nAMD: Neovascular age-related macular degeneration
PVC: Polypoidal choroidal vasculopathy
RAP: Retinal angiomatous proliferation
