# Supplementary material for: Higher plasma levels of complement C3a, C4a and C5a increase the risk of subretinal fibrosis in neovascular age-related macular degeneration: Complement activation in AMD
Source: Immun Ageing. 2016 Feb 16;13:4. doi: 10.1186/s12979-016-0060-5 (PMC4754842; doi:10.1186/s12979-016-0060-5)
Supplement: Additional file 1: Figure S1. — The relationship between the number of anti-VEGF injections and the concentration of complement fragments in the plasma. No correlation was observed between the number of intravitreal anti-VEGF injections and the plasma levels of C3a, C4a and C5a. (PDF 118 kb) [file 12979_2016_60_MOESM1_ESM.pdf]

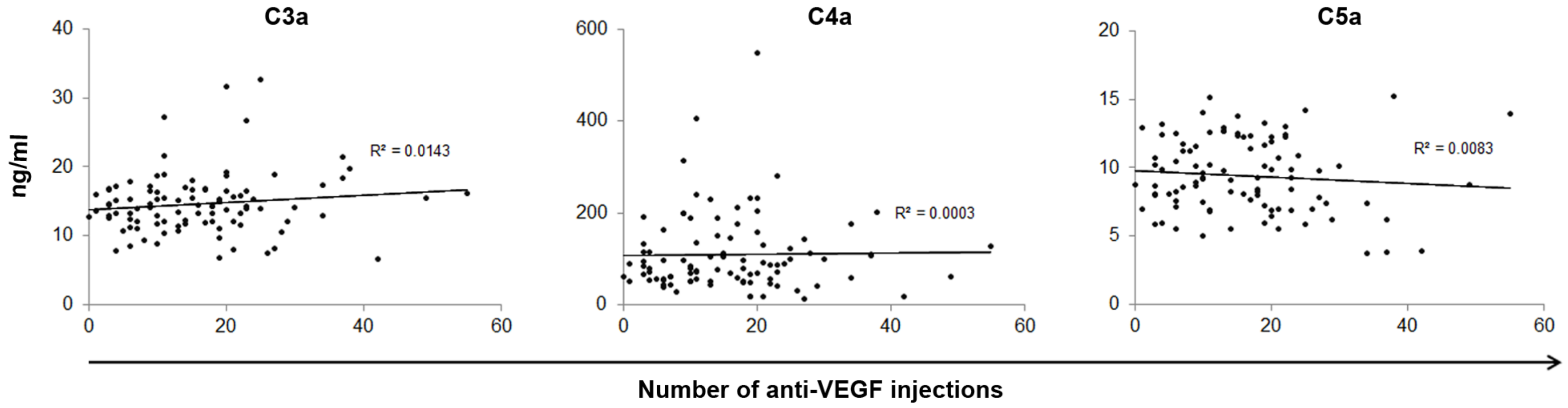

Supplemental Figure 1: The relationship between the number of anti-VEGF injections and the concentration of complement fragments in the plasma. No correlation was observed between the number of intravitreal anti-VEGF injections and the plasma levels of C3a, C4a and C5a.
